# Study on Filtration Performance of PVDF/PUL Composite Air Filtration Membrane Based on Far-Field Electrospinning

**DOI:** 10.3390/polym14163294

**Published:** 2022-08-12

**Authors:** Han Wang, Yiliang Bao, Xiuding Yang, Xingzi Lan, Jian Guo, Yiliang Pan, Weimin Huang, Linjun Tang, Zhifeng Luo, Bei Zhou, Jingsong Yao, Xun Chen

**Affiliations:** 1 State Key Laboratory of Precision Electronic Manufacturing Technology and Equipment, Guangdong University of Technology, Guangzhou 510006, China; 2 Foshan Nanofiberlabs Co., Ltd., Foshan 528225, China; 3 School of Mechanics and Astronautics, Nanyang Technological University, 50 Nanyang Avenue, Singapore 639798, Singapore

**Keywords:** electrospinning, biopolymer, composite structure, air filtration, nanofiber membrane

## Abstract

At present, the situation of air pollution is still serious, and research on air filtration is still crucial. For the nanofiber air filtration membrane, the diameter, porosity, tensile strength, and hydrophilicity of the nanofiber will affect the filtration performance and stability. In this paper, based on the far-field electrospinning process and the performance effect mechanism of the stacked structure fiber membrane, nanofiber membrane was prepared by selecting the environmental protection, degradable and pollution-free natural polysaccharide biopolymer pullulan, and polyvinylidene fluoride polymer with strong hydrophobicity and high impact strength. By combining two kinds of fiber membranes with different fiber diameter and porosity, a three-layer composite nanofiber membrane with better hydrophobicity, higher tensile strength, smaller fiber diameter, and better filtration performance was prepared. Performance characterization showed that this three-layer composite nanofiber membrane had excellent air permeability and filtration efficiency, and the filtration efficiency of particles above PM 2.5 reached 99.9%. This study also provides important reference values for the preparation of high-efficiency composite nanofiber filtration membrane.

## 1. Introduction

Anthropogenic air pollution places a heavy burden on public health in most countries. Particulate matter (PM) is a microscale air pollutant that causes 3.3 million premature deaths per year and has been identified as a significant risk factor for premature death [1,2]. Controlling air quality has therefore become a critical aspect of public health and sustainable human growth. Major indoor sources of PM include environmental tobacco smoke (ETS), cooking, home heating fuel burning, and incense burning [3]. Among the various air purification methods, the most common technique is PM filtration, which involves removing particulate matter from the air through a filtering medium. The air filtration process can be divided into two states, namely, steady state (the first state) and unsteady state (the second state). For the first state, the particle-capture efficiency and the filter-pressure drop through the filter material are immutable over a period of time and completely depend on the inherent characteristics of the air filter medium, particle properties, and airflow velocity. According to the classical filtration theory, the stable stage of fiber filtration can be further divided into the following five capturing mechanisms: interception, inertial impact, Brownian diffusion, electrostatic effect, and gravity effect [4,5]. Fibrous materials have been the most widely used materials because of their high porosity structure, which facilitates airflow while successfully removing particles. However, the traditional air filter still has problems, e.g., the filter material usually has such problems as small pore size and large thickness. Although the traditional filter material has high filtration efficiency, it also produces a large pressure drop, which leads to poor overall filtration performance of filters [6].

Electrospinning is one of the efficient methods for manufacturing ultrafine nanofiber membranes. It applies a high voltage (usually several kV) to the polymer solution (or molten polymer) to produce ultrafine fibers (from 2 µm down to a few nanometers) with a high surface area and surface charges [7]. The first observation of an electrospinning process for such purpose was in 1902 by J.F. Cooley, who patented the technique with the name of [8]. With the continuous development in recent years, electrospinning has gradually become an important part of the nanofabrication process. Nanofiber membranes produced by electrospinning have been used in various fields, such as drug delivery [9,10,11], wound healing [12,13,14], filtration and separation [15,16], tissue engineering [17,18,19], textiles [20,21] with food packing [22,23], sensors [24], battery separators [25] and so on. In terms of filtration application, the electrospinning fiber air filter membrane can be regarded as working in the stable filtration stage [26]. The fiber diameter has a great influence on the filtration efficiency. When the fiber diameter is less than 0.5 μm, the filtration efficiency of the fiber membrane can be effectively improved under the premise of constant pressure drop. The fiber diameter of general non-woven fabric is generally about 12 μm, while the diameter of submicron fiber can reach 250 nm, which is about two orders of magnitude different. Studies have shown that nanofiber air filters prepared by electrospinning technology have the advantages of smaller diameter, higher porosity, better permeability, larger specific surface area, and easier control of fiber composition and morphology, which can significantly improve filtration efficiency and service life [27]. Electrospinning nanofibers have wide application prospects in filtration. At present, electrospinning nanofibers are mainly applied in the field of filtration in air filtration and liquid filtration. Impurities are intercepted on the surface or inside the membrane to achieve the purpose of purification [28].

Polyvinylidene fluoride (PVDF) is a semicrystalline polymer polymerized from vinylidene fluoride monomer via a free-radical polymerization, and therefore has various molecular weights due to the different degrees of polymerization. Compared with other fluoropolymers, PVDF has a low melting point of 170 °C and a density of 1.77 kg/m^3^. The glass transition temperature of PVDF is about −35 °C, and it is typically 50–60% crystalline [29]. It has high mechanical strength, good chemical resistance, thermal stability, and excellent aging resistance, which are very important for its practical application in filtration membranes, and is also widely used in scientific research and industrial processes [30]. It also has similar morphology and structure to the surface of lotus leaf, with the characteristic of high water contact angle, oil repellent effect, excellent ash collection effect, and self-cleaning performance, and is widely used in electrospinning nanofiber membrane manufacturing [31].

Biopolymers are among the most investigated biodegradable materials for developing food packaging materials because of their remarkable properties, including renewability, abundance, non-toxicity, biodegradability, biocompatibility, and functionality [32]. Pullulan polysaccharides (pullulan, abbreviated PUL) is a natural linear biopolymer with excellent film-forming properties and is extensively used in developing food packaging materials. It has a chemical formula of (C_6_H_10_O_5_)*_n_* and is mainly composed of α-(1, 6) glycosidic bond connection of malt trisaccharide units and is connected by a α-(1, 4) glycosidic bond of three malt trisaccharide of glucose units, with the characteristics of being non-toxic, non-mutagenic and -carcinogenic, biodegradable, and edible [33]. Moreover, it is a non-hygroscopic polymer with considerable intermolecular mechanical strength and processing-film properties, which has a wide range of commercial and industrial applications in many fields, such as food science, healthcare, pharmaceuticals, and even in the field of lithography. Because of its strict linear structure, it is also very valuable in basic research [34]. In recent years, PUL has been widely used in electrospinning nanofiber membrane manufacturing, drug delivery [35], wound dressing [36], and food preservation [37], and people are also increasingly interested in pullulan polysaccharides.

Based on the advantages of nanofiber membrane in the preparation process, materials and fiber overlapping structure, this work innovatively used the composite manufacturing process to the interlayer-stack structure nanofiber membrane’s electrospinning. By using PUL, an environmentally friendly, degradable, and pollution-free natural polysaccharide biopolymer, and PVDF polymer material with strong hydrophobic properties and high impact strength, a nanofiber air filtration membrane with high tensile strength, hydrophobic properties, excellent air permeability, and filtration performance was prepared. It can keep high efficiency and lasting stability in various complex filtering situations. This study also provides important reference values for the subsequent preparation of high-efficiency composite nanofiber filtration membrane.

## 2. Materials and Methods

### 2.1. Materials

PVDF (average Mv~300,000) was imported from Arkema, Paris, France. PUL was bought from Shanghai Aladdin Biochemical Technology, Shanghai, China. N,N-dimethylformamide (DMF, analytical pure) was bought from Shanghai Aladdin Biochemical Technology, Shanghai, China. Glacial acetic acid (ACS, ≥99.7%) was bought from Shanghai Aladdin Biochemical Technology, Shanghai, China. Acetone (analytical pure AR, ≥99.5%) was bought from Guangzhou Chemical Reagent Factory, Guangzhou, China.

### 2.2. Preparation of Electrospinning Solution

Preparation of polyvinylidene fluoride (PVDF) electrospinning solution: appropriate proportions of PVDF powder were weighed and slowly added into a glass bottle containing 10 mL of organic solvent (DMF:Acetone = 7:3) and placed in a magnetic stirrer for stirring. During the stirring process, the PVDF electrospinning solution with mass fractions of 14%, 18%, and 22% was obtained after heating in a water bath at 60 °C for 4 h.

Preparation of pullulan polysaccharide (PUL) electrospinning solution: PUL powder with appropriate proportions was weighed and slowly added into 50% acetic acid aqueous solution in batches. The PUL electrospinning solution was stirred at normal atmospheric temperature for 4 h in a magnetic stirrer, and then the PUL solution with mass fraction of 16%, 20%, and 24% was obtained.

### 2.3. Preparation of Fiber Membrane

**Figure 1:**
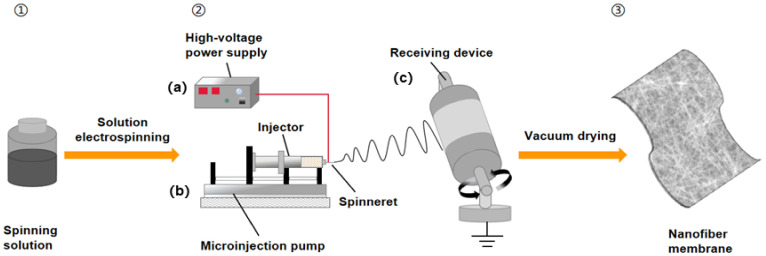
Schematic diagram of far-field electrospinning. **①** Spinning solution; **②** Electrospinning device (**a**) High voltage DC power supply; (**b**) Liquid supply device; (**c**) Collecting roller; **③** Electrospun nanofiber membrane.

The process of preparing nanofiber membrane by far-field electrospinning is shown in Figure 1. First, assemble the prepared polymer solution to the syringe pump shown in Figure 1b for pumping, and apply 0~30 kV high voltage to it through the high-voltage power supply shown in Figure 1a. The applied voltage induces charge movement in the polymer liquid. Under the influence of surface tension, the solution presents a similar falling shape. When the electrostatic repulsion of charged polymer liquid is higher than the surface tension, a Taylor cone will be formed, and the jet will be pulled into a smaller charged jet from the tip of the cone. If there is enough cohesion in the polymer liquid, a stable jet is sprayed from the Taylor cone to stretch the polymer chains and form uniform filaments. With the evaporation of solvent, the jet is pulled to the grounding collection roller, as shown in Figure 1c, in a spiral whip state, and by adjusting the distance between the roller and the syringe pump needle, the release paper attached to the collection roller can finally be collected to obtain a nanofiber membrane composed of micro- and nanofibers [38,39]. It is worth noting that the two forces inducing the formation of the Taylor cone are indirectly controlled by the flow rate and the applied voltage. Therefore, a good balance between the two parameters is conducive to the formation of a stable jet and the spinning of nanofiber membrane with better performance and morphology.

The syringe containing the spinning solution was equipped with a 20 G steel needle and was pumped at a matching flow rate using an injection pump (LSP02-1B, Baoding Longer Precision Pump, Baoding DiChuang Electronic Technology, Baoding, China). A voltage of 18 kV (Gamma high voltage, Ormond Beach, FL, USA) was applied and the distance between the needle and the collecting roller was 15 cm. The nanofiber membrane was collected on the release paper on the grounded collecting roller, and the electrospinning nanofiber membrane was vacuum-dried at 60 °C for 12 h to evaporate the residual solvent. During the whole experiment, the electrospinning condition was kept at 25 °C and the humidity was 60%. The film thickness was in the range of 0.1–0.15 mm.

### 2.4. Fiber Morphology Observation

After vacuum drying at 60 °C for a certain period of time, the morphology of the nanofiber membrane was observed by scanning electron microscopy (SEM, TM4000, Hitachi, Chiyoda District, Japan) at an accelerated voltage of 15 kV. In order to obtain better conductivity, all samples were sputtered with gold plating at 10 mA for 30 s before SEM observation. At least 100 fibers were measured using the ImageJ soft imaging system, and then the frequency distribution of the fiber diameter of the nanofiber membrane was obtained.

### 2.5. FTIR and XRD Analysis of Nanofiber Membrane

Fourier-transform infrared (FT-IR) spectra of the nanofiber membrane were obtained with an infrared spectrometer (Nicolet iS50 FTIR, Thermo Fisher Scientific (China), Shanghai, China) from 4000 to 400 cm^−1^. The sample was vacuum-dried before measurements were conducted.

The X-ray diffraction (XRD) patterns of nanofiber membrane were obtained at ambient temperature by step-scanning on an X-ray powder diffractometer (Ultima III, TX, USA) using monochromatic Cu Kα radiation (λ = 1.54 Å) in the range of 2θ = 5°–50° with a step size of 0.02° and a scanning rate of 5°/min. The sample was vacuum-dried before measurements were conducted.

### 2.6. Mechanical Performance Test

The tensile properties of the fiber membrane were tested using a tensile testing machine (CMT2000, SUST, Zhuhai, China). The nanofiber membrane was made into a sample of 8 cm × 1.5 cm and clamped on a movable fixture. Uniaxial tension was applied to the fiber membrane at a displacement rate of 5 mm/min using a tensile testing machine until failure. Before the test, measured the thickness and width of the fiber membrane with a Vernier caliper, and the fiber membrane was selected to test and then the average value of the results was obtained. Thickness is an important parameter to calculate the fracture strength of the fiber membrane, and the thickness of fiber membrane was 0.7 mm. During the tensile test, the initial stretch length of the fiber membrane was 50 mm, the pre-tension was 0.5 cN, and the sample strip width was 1.5 cm. Each sample was tested, and a stress-strain curve was recorded after the results were averaged.

### 2.7. Filtration Effect Test

There are two main parameters of air filtration performance evaluation—air filtration efficiency and air filtration pressure drop. Air filtration efficiency reflects the ability of an air filtration device to remove tiny particles.

The air filtration efficiency calculation formula is as follows:

(1) $$\eta \;=\;\left(1\;-\;\frac{N_{2}}{N_{1}}\right)\;\times \;100\%$$

where η is the air filtration efficiency, N_1_ represents the number of corresponding particles upstream of the air filtration membrane to be measured, and N_2_ represents the number of corresponding particles downstream of the air filtration membrane to be measured.

Air filtration pressure drop represents the obstruction ability of an air filter device to airflow. This is called “breathability”. The specific calculation formula is as follows:

(2) $$\Delta P\;=\;P_{1}\;-\;P_{2}$$

where ΔP represents the air filtration pressure drop, P_1_ represents the pressure in front of the air filter membrane to be measured, and P_2_ represents the pressure behind the air filter membrane to be measured.

Quality factor (QF) is a performance index which considers filtration efficiency and pressure drop comprehensively. It can be calculated by two parameters: filtration efficiency (η) and pressure drop (ΔP). It is often used as a balance indicator of filter performance. Therefore, the higher the quality factor of the material, the better the filtration performance. QF is defined as

(3) $$\mathrm{QF}\;=\;-\frac{\ln\left(1\;-\;\eta \right)}{\Delta P}$$

These parameters can be used to comprehensively measure the filtration performance of nanofiber membranes [26].

**Figure 2:**
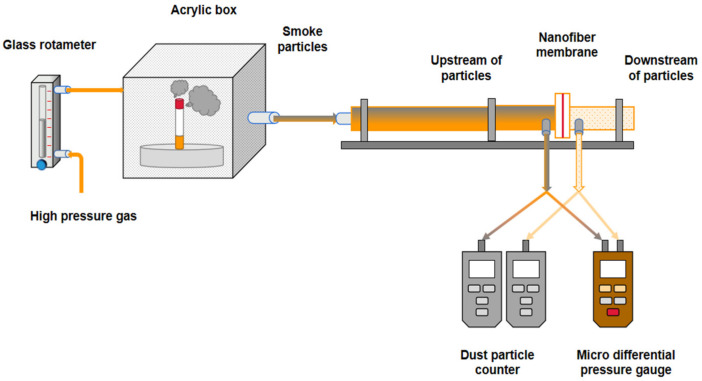
Composite testing device for filtration performance of nanofiber membrane.

Figure 2 shows the testing device for the filtration performance of electrospinning composite nanofiber membrane. A micro-differential pressure gauge (GM505, BENETECH) was used to measure the pressure drop before and after the flow through the nanofiber membrane at a specific surface velocity. The pressure drop was calculated by Formula (2).

The surface velocity is defined as the airflow through the filter per unit filter area. The airflow through the nanofiber membrane was generated by air compressor and controlled by a glass rotameter (LZB-4WB). The nanofiber membrane was cut into a circular sheet with a diameter of 30 mm and installed on the filter support. The particle matter was mainly generated in the acrylic box, and the smoke generated by the cigarette or mosquito repellent incense in the box was ignited by the airflow to the particle filtration and concentration measurement area. The number of PM 2.5 particles upstream and downstream was measured using a dust particle counter (MKS800, McCarthy), and then the filtration efficiency of the fiber membrane was calculated by Formula (1).

### 2.8. Water Contact Angle Test

Water contact angle was measured using a DCA 20 contact angle meter (Data Physics, Filderstadt, Germany). The electrospinning nanofiber membrane was fixed on the slide, and a drop of distilled water (5 μL) was added to the surface and the equilibrium time of droplet was 3 s before measurement. The water contact angle was tested in triplicate.

## 3. Results and Discussion

### 3.1. Effect of Solution Concentration on the Morphology of Nanofiber Membrane

#### 3.1.1. Exploration of the Optimal Concentration of PVDF Electrospinning Solution

In order to achieve the optimal morphology of nanofiber membrane, the optimal electrospinning concentration of PVDF electrospinning solution was investigated. In this section, the liquid supply speed was set as 1 mL/h, the total electrospinning time was 1 h, the voltage was 18 kV, the collection distance was 15 cm, and the speed of the collection roller was 0.2 r/s. Three kinds of PVDF nanofiber membranes were prepared by electrospinning with a concentration gradient of 14%, 18%, and 22%.

**Figure 3:**
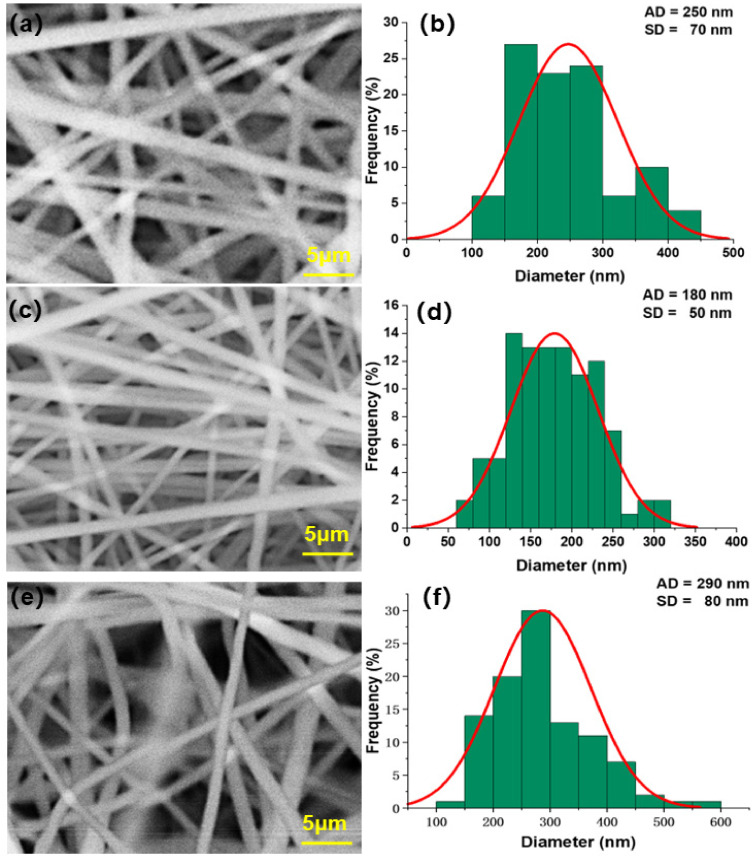
SEM morphology and fiber diameter frequency distribution of nanofiber membrane electrospun by PVDF solution at different concentrations. (**a**), (**c**) and (**e**) are SEM images of nanofiber membranes prepared by PVDF solution at 14%, 18% and 22%, respectively; (**b**), (**d**) and (**f**) are the fiber diameter frequency distributions of nanofiber membranes prepared by PVDF solution at 14%, 18% and 22% concentrations, respectively.

Figure 3a,c,e show the SEM morphology of nanofiber membrane electrospun by PVDF solution at the concentration of 14%, 18%, and 22%, respectively. Figure 3b,d,f shows the frequency distribution of fiber diameter of electrospun nanofiber membrane with PVDF solution at 14%, 18%, and 22% concentrations, respectively.

It can be seen from Figure 3a,b that when containing 14% PVDF, after 1 h electrospinning time, the nanofiber diameter of the fiber membrane is large, and the morphology distribution is uneven. The fiber distribution is in the range of 200–1400 nm, and most of the fiber diameters are 300–800 nm, accounting for 85% of the total. The mean fiber diameter is 570 nm, and the standard deviation is 210 nm.

It can be seen from Figure 3c,d that when 18% PVDF is contained, after 1 h electrospinning time, the overall morphology distribution of the nanofiber membrane tends to be uniform, and the fibers are distributed in the range of 50–550 nm, most of which are 150–300 nm in diameter, accounting for 70% of the total. The mean fiber diameter is 250 nm, and the standard deviation is 90 nm. The minimum fiber diameter can reach about 50 nm, which can greatly improve the interception effect of fiber membrane on particles.

According to Figure 3e,f, when containing 22% PVDF, after 1 h electrospinning time, the fiber diameter of the nanofiber membrane is evenly distributed, and the fibers are distributed in the range of 100–800 nm, most of which are 100–300 nm, accounting for 75% of the total. The mean fiber diameter is 270 nm, and the standard deviation is 120 nm.

This part of the experiment shows that the diameter of nanofibers prepared by pure PVDF solution decreased with the increase of solution concentration. The reason is that with the increase of PVDF content, the solution viscosity gradually increases, and the intermolecular interaction force also increases, so the spinning jet is more concentrated due to the viscous resistance constraint. Under the effect of reasonably increased electric field between the jet and the collecting roller, the jet can be drawn into more slender nanofibers under the traction force, which makes the nanofibers collected in the roller smaller in diameter and more concentrated in frequency distribution. According to the filtration theory, a higher filtration efficiency can be obtained by reducing the fiber size, but when the bulk density of the fiber remains unchanged, the pressure drop increases [40]. When the concentration of PVDF solution was 14%, 18%, and 22%, the diameter of fiber showed a decreasing trend. The reduction in fiber diameter helps to strengthen the interception effect of fiber membrane on finer particles. Although the pressure drop will also increase with the continuous stacking of fibers, from the perspective of comprehensive performance, it has little impact. According to the above factors, when PVDF content is 18%, the diameter and standard deviation of the electrospinning nanofiber membrane’s fiber is the minimum, and its filtration efficiency is also more advantageous than other concentrations.

#### 3.1.2. Exploration of the Optimal Concentration of PUL Electrospinning Solution

In order to achieve the optimal morphology of nanofiber membrane, the spinning concentration of pullulan polysaccharide (PUL) electrospinning solution was investigated. In this section, the liquid supply speed was set as 1 mL/h, the total electrospinning time was 1 h, the voltage was 18 kV, the collection distance was 15 cm, and the speed of the collection roller was 0.2 r/s. Three kinds of PUL nanofiber membranes were obtained by electrospinning with concentration gradients of 16%, 20%, and 24%, respectively.

**Figure 4:**
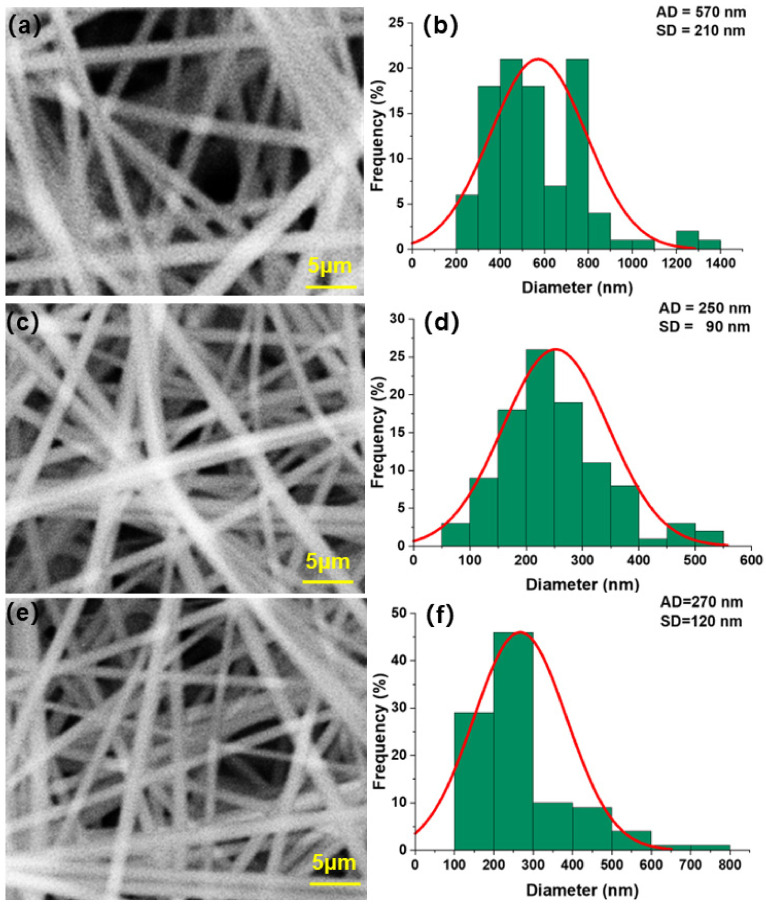
SEM morphology and fiber diameter frequency distribution of nanofiber membrane electrospun by PUL solution at different concentrations. (**a**), (**c**) and (**e**) are SEM images of nanofiber membranes prepared by PUL solution at 16%, 20% and 24%, respectively; (**b**), (**d**) and (**f**) are the fiber diameter frequency distributions of nanofiber membranes prepared by PUL solution at 16%, 20% and 24% concentrations, respectively.

Figure 4a,c,e show the SEM morphology of nanofiber membrane electrospun by PUL solution at concentrations of 16%, 20%, and 24%, respectively. Figure 4b,d,f show the frequency distribution of fiber diameter of electrospun nanofiber membrane with PUL solution at 16%, 20%, and 24% concentrations, respectively.

It can be seen from Figure 4a,b that when 16% PUL is contained, the fiber diameter of the fiber membrane is large and the morphology distribution is uneven. The fiber is distributed in the range of 100–450 nm, and most of the fiber diameters are 150–300 nm, accounting for 85% of the total proportion. The mean fiber diameter is 250 nm, and the standard deviation is 70 nm.

It can be seen from Figure 4c,d that when 20% PUL is contained, the overall morphology of the fiber membrane is evenly distributed and the fiber diameter is small and distributed in the range of 50–550 nm, most of which are 150–250 nm in diameter, accounting for 70% of the total. The mean fiber diameter is 180 nm, and the standard deviation is 50 nm.

It can be seen from Figure 4e,f that when 24% PUL is contained, the fiber diameter of the fiber membrane is evenly distributed and the fibers are distributed in the range of 100–600 nm, most of which are 150–400 nm in diameter, accounting for 75% of the total. The mean fiber diameter is 290 nm, and the standard deviation is 80 nm.

#### 3.1.3. Analysis of Pore Size of Electrospun Composite Nanofiber Membrane

**Figure 5:**
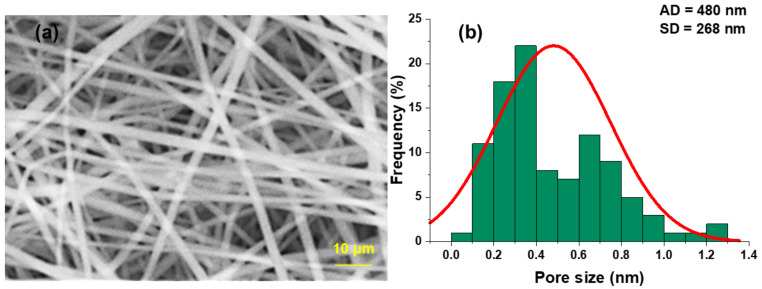
SEM morphology and pore size frequency distribution of electrospun composite structure nanofiber membrane. (**a**) SEM image of electrospun composite structure nanofiber membrane; (**b**) Pore size frequency distribution of electrospun composite structure nanofiber membrane.

It can be seen from Figure 5a,b that the pore size of the electrospun composite structure nanofiber membrane is evenly distributed, and the pore sizes are distributed in the range of 60–1300 nm, most of which are 100–800 nm in diameter, accounting for 87% of the total. The mean pore size is 480 nm, and the standard deviation is 268 nm.

This part of the experiment shows that the diameter of nanofibers prepared by pure PUL solution decreases gradually and then increases with the increase of solution concentration. The main reason is that with the increase in PUL content, the solution viscosity gradually increases, and the intermolecular interaction force also increases, and the spinning jet is more concentrated under the restriction of viscous resistance. Under the effect of reasonably increased electric field between the jet and the receiving roller, the jet can be drawn into more slender nanofibers under the traction force, which makes the nanofibers smaller in diameter and more concentrated in frequency distribution. When PUL concentration continues to increase, the jet is more constrained by viscous resistance, and it is difficult to disperse into smaller fibers under the influence of intermolecular force. Based on the above factors, when the PUL content is 20%, the fiber diameter and standard deviation of the electrospinning nanofiber membrane are the smallest.

According to these two parameters, electrospinning of the composite structure nanofiber membrane is carried out. The pore size distribution of the electrospun nanofiber membrane is relatively uniform, and the average pore size is less than 500 nm, which can effectively intercept particles above PM 0.5, and has good air filtration efficiency. With the extension of spinning time and the deepening of fiber stacking, its filtration performance will be further improved. Although the pressure drop will also increase slightly, it will have little impact on its comprehensive performance.

### 3.2. FTIR and XRD Analysis of Nanofiber Membrane

In order to further explore the properties of spinning nanofiber membranes, PUL, PVDF and composite structure nanofiber membranes were tested by FTIR and XRD.

**Figure 6:**
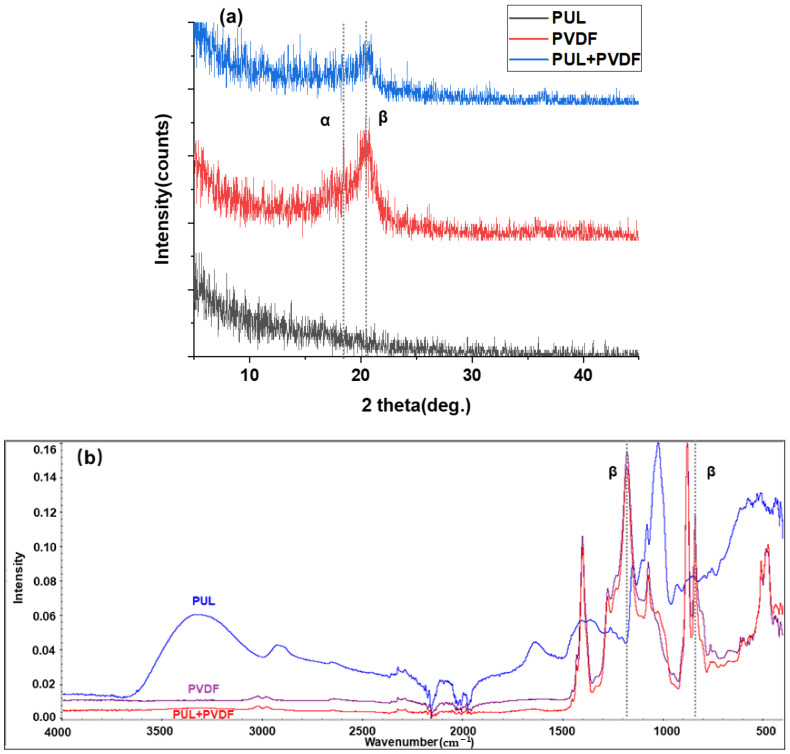
(**a**) XRD patterns and (**b**) FTIR spectra of the PUL, PVDF, and composite nanofiber membranes.

The crystal phase of the PUL, PVDF, and composite nanofiber membrane was examined using X-ray diffraction (XRD) and Fourier transform infrared spectroscopy (FTIR). As shown in Figure 6a, the XRD curves show a typical β crystal phase peak at around 2θ = 20.44°, which is assigned to the total diffraction in (110) and (200) planes. The peak at 18.42° corresponds to the reflection of the (020) plane of the α phase.

Figure 6b show the FTIR spectra of the PUL, PVDF, and composite nanofiber membrane. The vibration bands at 840 cm^−1^ and 1179 cm^−1^ were assigned to the absorption bands of all-trans configuration of the β crystal phase [41].

Observing the XRD and FTIR data of three kinds of nanofiber membranes, it can be found that the composite structure nanofiber membrane has a crystalline peak similar to PVDF nanofiber membrane at 20.44° and vibration bands similar to PVDF nanofiber membrane at 840 cm^−1^ and 1179 cm^−1^, although the strength is slightly lower, and no similar characteristic peak or vibration bands of PUL nanofiber membrane are observed. It can be considered that in the composite structure nanofiber membrane, the outer PVDF nanofiber membrane can well cover the inner PUL nanofiber membrane, and there is a good overlap between the fibers, and the composite structure nanofiber membrane can not only maintain the high heat and hydrophobic strength of PVDF nanofiber membrane, but also better play the role of nanofiber membrane.

### 3.3. Effect of Composite Structure on Mechanical Properties of Nanofiber Membrane

#### 3.3.1. Effect of Three-Fiber Membrane Types on Mechanical Properties of Nanofiber Membrane

In order to explore the influence of composite structure on the mechanical properties of nanofiber membrane, the mechanical properties of pure PVDF nanofiber membrane, pure PUL nanofiber membrane, and interlayer-stack structure nanofiber membrane were tested.

**Figure 7:**
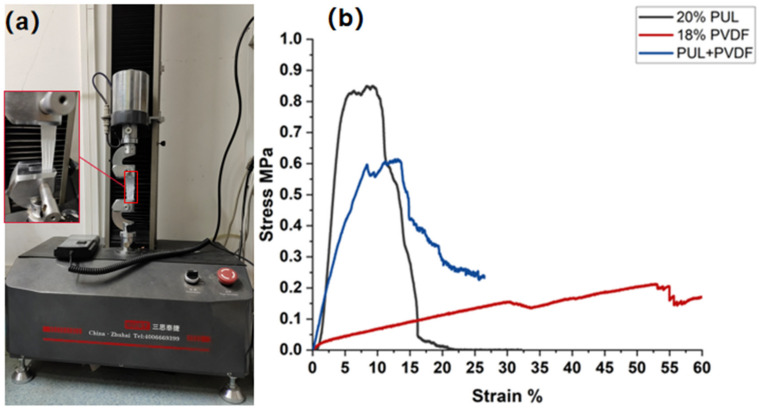
Microcomputer-controlled electronic universal testing machine and fiber stress-strain diagram of nanofiber membrane. (**a**) Microcomputer-controlled electronic universal testing machine; (**b**) fiber stress-strain diagrams of three types of nanofiber membranes.

It can be seen from Figure 7b that, by controlling the total electrospinning time as 1 h, the experimental data of pure PVDF fiber membrane, pure PUL fiber membrane, and interlayer-stack nanofiber membrane were compared. It was found that the tensile strength of interlayer-stack nanofiber membrane spun under group B was between the pure PVDF nanofiber membrane and the pure PUL nanofiber membrane. It was 65.9% higher compared with pure PVDF nanofiber membrane and 38.2% lower than pure PUL nanofiber membrane. Therefore, it can be explained that the nanofiber membrane spun with interlayer-stack structure has better mechanical properties and can maintain stable filtration performance under high airflow.

#### 3.3.2. Effect of Interlayer-Stack Structure on Mechanical Properties of Nanofiber Membrane under Three Spinning Time Ratios

The stacked structure of fibers has great influence on the filtration performance of electrospinning membrane. To remove particles from polluting gases, the particles must collide with the fibers and remain stationary. Sufficient fine fibers, compact stacked structure, high thickness, and significant electrostatic effect all contribute to effective particle capture [23]. In the process of improving the filtration performance of the nanofiber membrane, it is found that the fiber with the interlayer-stack structure—a kind of three-layer composite structure where one material serves as the upper and lower layers and another material serves as the inner layer—can improve the overlapping layers of fibers between the nanofiber membrane layers, and the porosity and permeability of the nanofiber membrane are significantly improved. This is a way to increase the specific surface area of the nanofiber membrane in addition to reducing the diameter of the fiber, and it can increase the probability of particles colliding with fibers. In addition, when particles collide with a single fiber, the shear flow of air near the fiber will drive the particles to move. Fibers in the interlayer-stack structure can rely on its interlaced fiber network to continuously use the friction force on the fiber surface to consume the kinetic energy of particles and achieve the effect of retaining particles [26]. Therefore, fabrication of interlayer-stack nanofiber membrane can effectively improve the particle interception ability and enhance its filtration efficiency.

Based on the above principle, the preparation process of PVDF/PUL/PVDF interlayer-stack nanofiber membrane was designed. The basic process is to spin a layer of PVDF nanofiber membrane on the release paper, and spin it for t_1_ time under suitable conditions. PUL nanofiber membrane continued to be spun on the PVDF fiber membrane surface, and t_2_ time was spun under suitable conditions. PVDF nanofiber membranes were further spun on the surface under suitable conditions for t_3_. Finally, a PVDF/PUL/PVDF nanofiber membrane with interlayer-stack structure was obtained. In order to explore the optimal combination ratio, the spinning experiments with three spinning time ratios will be designed, namely, by controlling the total electrospinning time as 1 h, the nanofiber membrane with PVDF/PUL/PVDF interlayer-stack structure would be spun according to the following three spinning time ratios: A (t_1_:t_2_:t_3_ = 1:1:1), B (t_1_:t_2_:t_3_ = 1:2:1) and C (t_1_:t_2_:t_3_ = 1:4:1), and its mechanical properties and filtration efficiency would be characterized.

**Figure 8:**
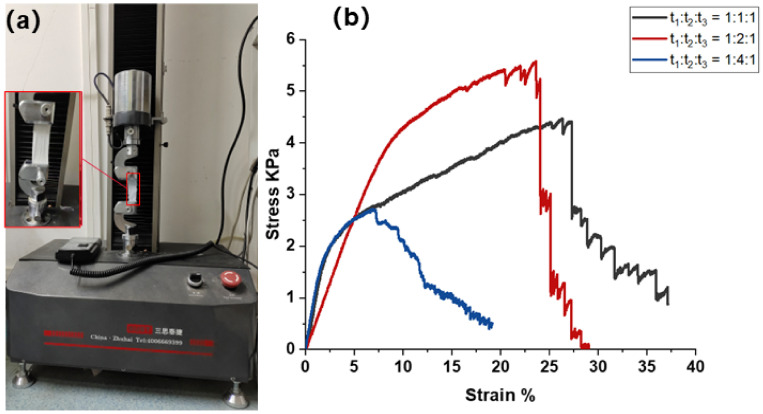
Microcomputer-controlled electronic universal testing machine and fiber stress-strain diagram of nanofiber membrane. (**a**) Microcomputer controlled electronic universal testing machine; (**b**) fiber stress-strain diagram of composite structure nanofiber membrane.

It can be seen from Figure 8b that in the three groups of experiments, the tensile strength of the nanofiber membrane electrospun under the condition of group B (t_1_:t_2_:t_3_ = 1:2:1) with 1 h total electrospinning time is the best, and is 24.7% and 104.8% higher than that of group A and C respectively. Therefore, under the experimental conditions of group B, the nanofiber membrane spun with the interlayer-stack structure has better mechanical properties, can adapt to the complex and changeable air filtration environment, and maintain lasting filtration performance under high air flow.

### 3.4. Effect of Composite Structure on Filtration Performance of Nanofiber Membrane

#### 3.4.1. Effect of Three Types of Fiber Membrane on Filtration Performance of Nanofiber Membrane

Under the airflow flow rate of 1 L/min, the number of particles upstream and downstream of the three groups of composite structure nanofiber membranes spun under pure PUL, pure PVDF, and group B conditions were counted and the pressure drop was measured.

**Figure 9:**
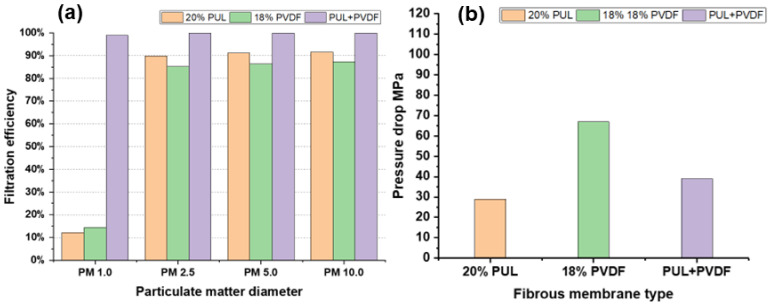
Particle filtration efficiency and pressure drop data of three types of nanofiber membranes. (**a**) Comparison diagram of filtration efficiency of three types of nanofiber membranes for different particles; (**b**) pressure drop comparison diagram of three types of nanofiber membranes.

According to Figure 9, the filtration efficiency of pure PVDF nanofiber membrane for PM 10.0, PM 5.0, PM 2.5, and PM 1.0 particles is 12.1%, 89.93%, 91.13%, and 91.58%, respectively, and the corresponding QF values are 0.0044, 0.0791, 0.0835, and 0.0853, respectively. The pressure drop of the nanofiber membrane is 29 Pa.

The filtration efficiency of pure PUL nanofiber membrane for PM 10.0, PM 5.0, PM 2.5, and PM 1.0 particles is 15.79%, 93.73%, 95.06%, and 95.98%, and corresponding QF values are 0.0025, 0.0413, 0.0448, and 0.0479, respectively. The pressure drop of the membrane is 67 Pa.

The filtration efficiency of composite nanofiber membrane for PM 10.0, PM 5.0, PM 2.5, and PM 1.0 particles is 99.98%, 99.94%, 98.90%, and 98.52%, and the corresponding QF values are 0.2183, 0.1902, 0.1156, and 0.1080, respectively. The pressure drop of the fiber membrane is 39 Pa.

Comprehensive comparison shows that compared with pure PVDF and pure PUL nanofiber membrane, the composite nanofiber membrane has higher QF value, excellent filtration performance for PM 10.0–PM 1.0 particles, and good air permeability.

#### 3.4.2. Effect of Interlayer-Stack Structure on Filtration Performance of Fiber Membrane under Three Spinning Time Ratios

**Figure 10:**
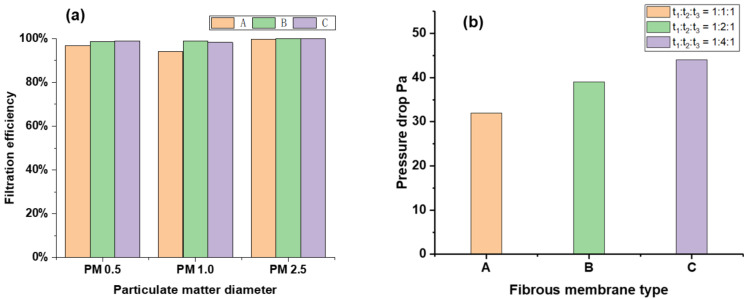
Filtration efficiency and pressure drop data of electrospinning nanofiber membrane particles under three spinning time ratios conditions A, B, and C. (**a**) Filtration efficiency of three composite nanofiber membranes for different particles; (**b**) Pressure drop of three composite nanofiber membranes.

It can be seen from Figure 10 that under the condition of air flow of 1 L/min, the upstream and downstream particle quantity statistics and pressure drop values of fiber membranes spun under the conditions of group A, B, and C are measured.

Under the condition of group A with 1 h total electrospinning time, the filtration efficiency of the electrospinning nanofiber membrane for PM 2.5, PM 1.0, and PM 0.5 particles was 99.67%, 94.08%, and 96.74%, respectively, and the corresponding QF values were 0.088, 0.1070 and 0.1781, respectively. The pressure drop of the nanofiber membrane was 32 Pa.

Under the condition of group B with 1 h total electrospinning time, the filtration efficiency of the electrospinning nanofiber membrane on PM 2.5, PM 1.0, and PM 0.5 particles was 99.94%, 98.90%, and 98.52%, and corresponding QF values were 0.1156, 0.1080, and 0.1916, respectively. The pressure drop of the nanofiber membrane was 39 Pa.

Under the condition of group C with 1 h total electrospinning time, the filtration efficiency of the electrospinning nanofiber membrane for PM 2.5, PM 1.0, and PM 0.5 particles was 99.80%, 98.34%, and 98.87%, and the corresponding QF values were 0.093, 0.1019, and 0.1414, respectively. The pressure drop of the nanofiber membrane was 44 Pa.

Through comprehensive comparison, it can be concluded that the interlayer-stack structure nanofiber membrane spun under group B with 1 h total electrospinning time has the highest overall QF value, excellent filtration performance, and good air permeability. Because its outer layer is PVDF nanofiber membrane with high mechanical strength and strong thermal stability, combined with PUL nanofiber membrane with small fiber diameter and high porosity in the inner layer, it can achieve more efficient and comprehensive interception effect for finer particles, and can adapt to more complex and diverse filtration scenarios.

### 3.5. Hydrophobic Performance Evaluation and Mechanism Analysis of Composite Nanofiber Membrane

The water contact angle can be defined as the included angle between the tangent line and the contact point line formed at the three phase contact points on both sides of solid–liquid–gas after the droplet forms a Young–Laplace arc on the solid surface [42].

**Figure 11:**
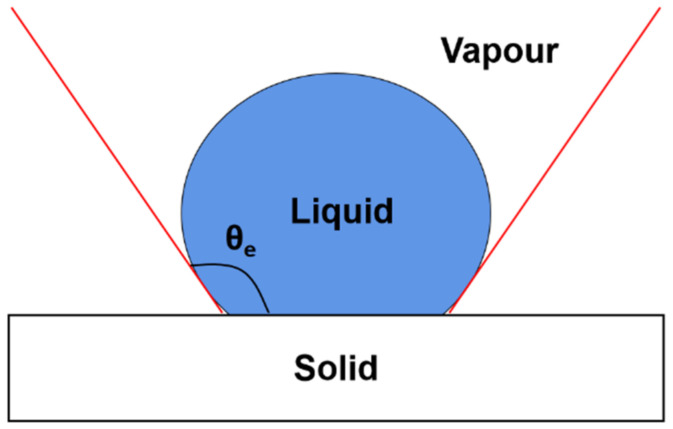
Schematic diagram of water contact angle.

As shown in Figure 11, if θ_e_ < 90°, the sample surface is hydrophilic, that is, the liquid is easier to wet the solid, and the smaller the angle of it, the better the wettability. If the contact angle θ_e_ is less than 10°, it is called superhydrophilic. In actual use, contact angle below 5° can have antifogging effect; If θ_e_ > 90°, the solid surface is hydrophobic, that is, the liquid cannot easily wet the solid and move on the surface. If the contact angle is θ_e_ > 150°, is called superhydrophobic.

**Figure 12:**
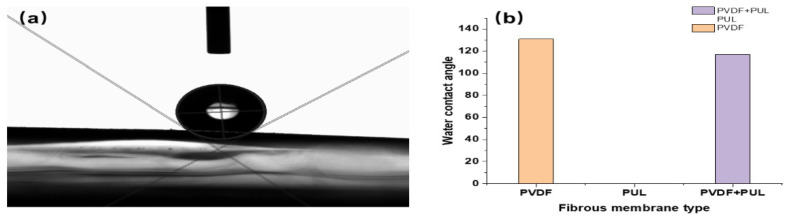
Water contact angle measurement of nanofiber membrane with three structures. (**a**) Water contact angle measurement; (**b**) Water contact angle comparison diagram of three types of nanofiber membranes.

According to Figure 12, it can be concluded that the average static water CA of PVDF fiber membrane is 131°, with good hydrophobicity. This is attributed to PVDF nanofiber membrane with higher surface roughness and lower surface energy, which makes it have excellent surface hydrophobicity. At the same time, it can be concluded that the average static water CA of PUL nanofiber membrane is 0°, with good wettability. However, due to its fine fiber diameter and high porosity, it has better filtration performance for small particles, and it is easier to adhere them on the membrane surface to achieve the interception effect. In addition, it can be concluded that the average static water CA of interlayer-stack structure nanofiber membrane is 117°, indicating that it has good hydrophobicity. This is attributed to the excellent surface hydrophobicity of PVDF nanofiber membrane, which enables it to maintain good filtration performance in high-humidity scenarios.

## 4. Discussion

In this paper, firstly, the far-field electrospinning technology, which is widely used because of its simple operation, wide application range and relatively high production efficiency, was used to prepare the nanofiber membrane. It is different from the traditional molding processes, such as stretching method, template synthesis, self-assembly, microphase separation, etc., the electrospun nanofiber membrane has better comprehensive performance, which cannot only combine the excellent properties of the polymer solution used, such as environmental protection, health, non-toxicity, and non-polluting, its material is cheap, easy to be degraded, etc. It can also better combine the characteristics of the nanofiber membrane electrospun by the corresponding polymer solution, such as heat resistance and hydrophobicity, high strength and toughness, small pore size and low pressure drop, and even further improve and even obtain new usable properties on this basis. Secondly, in the principle of proportional distribution of spinning time in the process of far-field electrospinning, the far-field electrospinning of PVDF and PUL polymer solution was carried out, and according to the characteristics of the fiber membrane spun by these two solutions, the fibers of the two were stacked step by step, with PVDF nanofiber membrane as the outer layer and PUL nanofiber membrane as the inner layer. By controlling the spinning sequence and time ratio of the two, finally, a novel sandwich-stack structure nanofiber film is formed. It has the advantages of heat resistance, hydrophobicity, high strength and toughness, fine fiber, small pore size, environmental protection, non-toxicity, and degradability. When applied to air filtration, it not only maintains stable performance in complex filtration scenarios but also further improves the interception ability of air particles and the efficiency of air filtration.

Through a series of characterizations of the composite structure nanofiber membrane, such as SEM, FTIR, XRD, mechanical property test, water contact angle test, particle filtration efficiency test, and pressure drop test, when the fiber diameter can reach the minimum, PVDF solution concentration is 18%, and the average fiber diameter is 250 nm, and when the fiber diameter can reach the minimum, PUL solution concentration is 20%, and the average fiber diameter is 180 nm. In addition, the composite structure nanofiber membrane’s average pore size is 480 nm, the tensile strength is 0.67 MPa (the total spinning time of this experiment is 1 h). Compared with similar literature, the tensile strength of PUL nanofiber membrane can reach 7.5 MPa, and that of PVDF nanofiber membrane can reach 14.8 MPa [43,44]. With the extension of electrospinning time, the tensile strength of nanofiber membrane can be further strengthened. In the XRD pattern, at 2θ = 20.44°, it is similar to PVDF nanofiber membrane β Crystal phase peak, and in the FTIR spectrum, wave numbers 840 cm^−1^ and 1179 cm^−1^ can be observed with PVDF nanofiber membrane having similar β phase. Its water contact angle is 117°. For particles PM 0.5, PM 1.0, and above PM 2.5, the filtration efficiency can reach 98.52%, 98.90%, and 99.96%, and the air pressure drop is 39 Pa.

This composite structure nanofiber membrane is compared with PAN/β-cyclodextrin (β-CD) composite nanofiber filter membrane (pressure drop 45 Pa, PM 2.5 filtration efficiency 99%), CS/PEO@MOF-5 composite nanofiber membrane (pressure drop more than 100 Pa, PM 2.5 filtration efficiency 99.95%), zein functionalized cotton fibers (Z-CoF)/zein electrospun nanofibers (ZNF) double-layered air filtration membrane (pressure drop 112.5 Pa, filtration efficiency 99%) [45], PUL/PVDF interlayer-stack structure nanofiber membrane has higher filtration efficiency and lower pressure drop, and has the advantages of heat resistance, hydrophobicity, and small pore size. This makes its application in filtering scenarios more adaptive and stable, and can be applied to richer filtering scenarios and maintain better filtering performance.

## 5. Conclusions

In this paper, based on the parameter optimization strategy of far-field electrospinning process, combined with the preparation process of composite structure nanofiber membrane, the nanofiber filtration membrane with greatly improved filtration efficiency was prepared, and its comprehensive performance was evaluated, including further discussion of filtration mechanism. On the premise of synthesizing its mechanical properties, the influence of preparation parameters on the performance of nanofiber filtration membrane was studied. The comprehensive comparative experiment showed that the formula of electrospinning solution was PVDF spinning solution (18%) and PUL electrospinning solution (20%), and according to the influence mechanism of fiber stacking structure on the comprehensive performance of nanofiber membrane, the electrospinning time ratio t_1_:t_2_:t_3_ = 1:2:1 with 1 h total electrospinning time was set for composite structure electrospinning, and the obtained interlayer-stack structure nanofiber membrane, It can integrate the excellent tensile strength and hydrophobic properties (CA = 117°) of the nanofiber membrane spun by the two materials, so that it can adapt to more complex filtration environments. Through the composite of two kinds of nanofiber membranes in fiber diameter and porosity with average pore size of 480 nm, the filtration performance of nanofiber membrane is better than that of single material. The filtration efficiency can reach 99.96% for particles above PM 2.5, 98.90% and 98.52% for particles above PM 1.0 and PM 0.5, and the pressure drop of the membrane can reach 39 Pa, with good air permeability. Also, according to the FTIR and XRD analysis, it can be seen that the outer PVDF nanofiber membrane can well cover the inner PUL nanofiber membrane, which has a good composite structure. In addition, the selected PUL material is a natural polysaccharide biopolymer, which degradable, pollution-free, and environmentally friendly. The research also provide important reference values for the preparation of high-efficiency nanofiber filter membranes with composite structure.

## Data Availability

The data that support the findings of this study are available from the corresponding author upon reasonable request.
